# Radiomic Characterization and Automated Classification of Drusen Substructure Phenotype Associated with High-Risk Dry Age-Related Macular Degeneration

**DOI:** 10.3390/diagnostics15202594

**Published:** 2025-10-15

**Authors:** Scott W. Perkins, Neal Shah, Jon Whitney, Karen Matar, Hannah J. Yu, Charles C. Wykoff, Justis P. Ehlers

**Affiliations:** 1 Cleveland Clinic Lerner College of Medicine of Case Western Reserve University, Cleveland, OH 44195, USA; 2 The Tony and Leona Campane Center for Excellence in Image-Guided Surgery and Advanced Imaging Research, Cole Eye Institute, Cleveland Clinic, Cleveland, OH 44195, USA; 3 Retina Consultants of Texas, Houston, TX 77090, USA; hannah.yu@duke.edu (H.J.Y.);; 4 Vitreoretinal Service, Cole Eye Institute, Cleveland Clinic, Cleveland, OH 44195, USA

**Keywords:** drusen, optical coherence tomography-reflective drusen substructures, radiomics, age-related macular degeneration, geographic atrophy

## Abstract

**Background/Objectives**: Optical coherence tomography (OCT)-reflective drusen substructures (ODSs) are associated with the conversion of intermediate AMD to geographic atrophy (GA). However, ODSs must be manually identified, a laborious process introducing bias and variation. This study proposes objective radiomic metrics of drusen phenotypes and validates them for the prediction of GA development and GA growth rate. **Methods**: A total of 104 drusen with high-reflective cores (H-type), 105 with low-reflective cores (L-type), 129 conical drusen (C-type), and 101 normal drusen (N-type) were segmented from OCT images. Radiomic features were extracted from these drusen, and the most important features for drusen classification were extracted from the retinal pigment epithelium–Bruch’s membrane compartment of 743 OCT scans of eyes with dry AMD and used to predict GA conversion and fast growth. **Results**: Radiomic features classified drusen phenotypes with AUC = 0.87–0.95. H-type drusen have a higher reflectivity, greater variation in reflectivity, and coarser texture (*p* < 0.001). L-type drusen have a lower reflectivity and greater variation in reflectivity (*p* < 0.0001). C-type drusen have a less spherical shape and more disordered internal reflectivity (*p* < 0.001). N-type drusen have a more spherical shape and more uniform internal reflectivity (*p* < 0.001). These radiomic features predict the conversion from intermediate AMD to GA and top-quartile GA growth rate with AUC = 0.59–0.74 at years 1–3. **Conclusions**: These results demonstrate the potential of clinical phenotype-grounded radiomics for objective automated drusen analysis, GA risk stratification, and clinical prediction.

## 1. Introduction

Dry age-related macular degeneration (AMD) is one of the most common (~288 million cases by 2040) causes of irreversible blindness and has a variable course [1,2]. Biomarkers on fundus exams and retinal imaging are important for disease staging and prognostication [3]. A qualitative analysis of biomarkers has long been used to clinically evaluate AMD, but quantitative biomarker analysis is increasingly important for clinical trial design, precision medicine applications, and improved clinical prognostication [3,4,5]. Spectral-domain optical coherence tomography (OCT) imaging enables a cross-sectional retinal visualization and biomarker identification with a high resolution [6]. Such biomarkers can be quantified by human graders or automated systems, such as machine learning-based platforms that improve efficiency and scalability for research and clinical trial applications [4,7]. Such machine learning methods have been applied to a variety of retinal compartments and biomarkers in dry AMD, including ellipsoid zone loss, which was recently made an FDA-approvable clinical trial endpoint [4,7]. However, despite many advances in the machine learning-based quantification of OCT imaging biomarkers in dry AMD, this technology has not been widely applied to an analysis of the drusen phenotype, one of the most classic and important biomarkers of AMD.

Drusen are compositionally varied lipid and protein deposits in the macula between the retinal pigment epithelium and Bruch’s membrane [8,9]. Drusen are a hallmark of early and intermediate AMD, and the presence of large drusen (>125 um) is a classic predictor of progression to geographic atrophy [5]. Cross-sectional OCT imaging of drusen shows OCT-reflective drusen substructures (ODSs) that further confer a risk of geographic atrophy (GA) in intermediate AMD eyes [10]. ODS phenotypes include low-reflective cores (L-Type), high-internal-reflective cores (H-Type), and homogeneous internal reflectivity with no ODSs (N-Type) [10]. Currently, ODSs must be manually identified by a trained grader, a laborious process that introduces potential bias and variation [10]. An automated and machine learning-based analysis of ODSs could enable an automated drusen analysis for drusen burden quantification and AMD clinical course prediction.

An automated ODS analysis would require an algorithmic quantification of drusen shape, reflectivity, and texture, as these characteristics distinguish ODS phenotypes [10]. Radiomic analysis is a set of mathematical techniques that quantifies pixel intensity, texture, and shape patterns in images and image regions that are biologically relevant [11]. The resulting radiomic metrics can then be inputted into machine learning or other predictive models for phenotype classification and disease prognostication [11]. Radiomic techniques first showed utility in cancer biology for disease staging and prognostication based on histologic and radiologic images [12,13]. Recent ophthalmological studies predicted sub-foveal GA in dry AMD using radiomics of the EZ-RPE and RPE-BM compartments, treatment durability in retinal vein occlusion and diabetic macular edema using radiomics of retinal fluid and tissue compartments, and treatment response and durability in neovascular AMD using radiomics of retinal fluid, tissue, and subretinal hyperreflective material [14,15,16,17].

While previous studies used radiomic features to predict retinal disease progression, these studies did not investigate drusen specifically, and used broad compartmental screening approaches to find predictive features without specifying features that reflect a known clinical phenotype. As machine learning-based systems become more prevalent in medical imaging, the need for interpretable and understandable systems has been identified, as less interpretable systems can give unexpected outputs that may hinder accuracy and physician and patient trust [18]. This study builds on previous work by testing the feasibility of the radiomics-derived differentiation of drusen substructure phenotypes to further understand the assessment in evaluating the risk for disease progression, potentially demonstrating the utility of clinically grounded radiomic features for understandable and trustworthy automated prediction in dry AMD.

## 2. Materials and Methods

**Figure 1:**
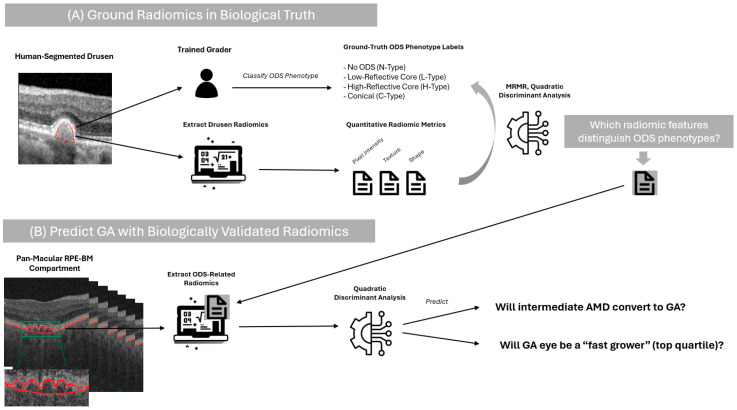
Diagram of study methods.

This was an IRB-approved retrospective image analysis study. The study was conducted in accordance with the Declaration of Helsinki and approved by the Institutional Review Board of Cole Eye Institute, Cleveland Clinic (Approval code 14-1527; approval date: original date, 12 September 2014; most recent renewal date, 12 September 2024). Guidelines for retrospective studies and good clinical practice were followed, including adherence to the Declaration of Helinski, International Conference on Harmonization of Technical Requirements of Pharmaceuticals for Human Use E6, applicable Food and Drug Administration regulations, and the Health Insurance Portability and Accountability Act. Given the retrospective nature of this study, limited to anonymized imaging data, patient consent was waived by the institutional review board. The workflow for this analysis is depicted in Figure 1.

### 2.1. Drusen Training Dataset

**Figure 2:**
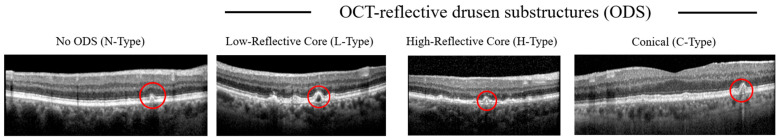
Illustrative examples of drusen substructure phenotypes.

To develop a dataset of drusen with ODS phenotypes for radiomic extraction, OCT B-scans were manually graded for the presence of ODSs using two independent graders with a final arbitrator, the same grading framework as Veerappan et al. [10]. A total of 104 N-type, 105 L-type, 101 H-type, and 129 C-type drusen were manually segmented from OCT scans of 75 patients from Cole Eye Institute (Cirrus scans, 31 patients) and Retina Consultants of Texas (Spectralis scans, 44 patients). ODSs were defined in a similar manner as described previously in the literature: L-type had a focal, well-circumscribed sub-volume of distinct hypo-reflectivity within the druse relative to the surrounding druse material, not contiguous with the RPE or BM and with size > 2 pixels; H-type had a focal, well-circumscribed sub-volume of distinct hyper-reflectivity within the druse relative to the surrounding druse material, not contiguous with the RPE or BM and size > 2 pixels; C-type had a conical shape—in contrast to the definition of Veerappan et al., debris criteria were not included, as very few conical-shaped drusen with debris were found in this dataset—a notable difference not previously reported; N-type was defined as a druse of homogeneous reflectivity [10]. Pixel-level thresholds were used for consistency with Veerappan et al., as well as for consistency across scans as image resolution was on the order of microns per pixel. Representative examples are shown in Figure 2. Although split reflective drusen were identified by Veerappan et al., only one druse of this type was found in these datasets, so this subtype was excluded from the analysis.

### 2.2. Radiomics Extraction from Drusen

For each segmented druse, 104 radiomic features were extracted from the segmented mask quantifying drusen pixel intensity, shape, and texture using previously reported feature definitions [19].

### 2.3. Selection of Radiomic Features Best Discriminating ODS Phenotype

For each ODS type *T*, the following methods were used to select the features best distinguishing *T*. For *k* in range 0–20, the top *k* most important features *F* were selected by the minimum redundancy maximum relevance method [20]. *F* were then used to train and validate a quadratic discriminant analysis classifier in a cross-validated schema, wherein repeated 80/20 train/validation splits were taken with varying random initiation states, yielding a mean AUC. The *k* and *F* yielding the greatest mean AUC were then taken to be the most important discriminating features for ODS type *T*. While this method resulted in drusen from the same eye being included in both the training and validation sets, model bias was avoided, as the ODS definitions were consistent across eyes, and the appearance of a given ODS type was not unique to a given eye. Furthermore, the most important features were selected using a dataset of individual drusen radiomics but tested on a separate dataset of RPE-BM radiomics as described below to limit bias.

### 2.4. Analysis of Best-Discriminating Features

The performance of best features in discriminating each ODS phenotype was assessed by training and testing a quadratic discriminant analysis classifier with the cross-validation methods described above. For each best discriminating feature, the variation in that feature across ODS phenotypes was tested using a one-way ANOVA (with Welch’s ANOVA used in cases of variance non-homogeneity as determined by the Levene test) under the central limit theorem (*n* > 30 in each group satisfying the normality assumption). Differences in radiomic values between individual ODS phenotypes were assessed using post hoc Tukey’s T-Tests. An alpha level of 0.05 was selected and corrected for multiple comparisons using Bonferroni’s correction, yielding a *p*-value cutoff of 0.00035 after correcting for 144 comparisons (6 post hoc comparisons per feature across 24 most important features).

### 2.5. Radiomics Extraction from the RPE-BM Compartment

After selection and analysis of features best discriminating ODS phenotype using the dataset of individual drusen radiomics, the following methods were used to assess the utility of these ODS-discriminating radiomic features in predicting GA on a separate dataset of radiomics extracted from the entire RPE-BM compartment. The RPE and BM of SD-OCT cubes of 743 eyes from 743 patients with intermediate AMD (513 eyes) or GA (220 eyes) were segmented by a previously reported deep learning system [7] and corrected by certified human graders. Using this segmentation, a mask of the RPE-BM compartment was created for each B-scan. Radiomic features previously found to be best-discriminating of ODS phenotypes were then extracted from these RPE-BM compartments, and the macular-cube-level median, kurtosis, skewness, and variance of each feature were calculated.

### 2.6. Prediction of GA Conversion and Fast Growth with ODS-Related Radiomic Features

For each study eye, macular-cube-level RPE-BM compartment radiomic features obtained from the above methods were used to train a quadratic discriminant analysis classifier predicting either conversion to GA (for intermediate AMD eyes) or fast GA progression (square-root GA growth rate in the top quartile of all study eyes with GA) at years 1, 2, and 3 after baseline. Data from the same eye were not included in both the training and testing sets to avoid model bias. The fast GA prediction analysis was repeated on a subset of data only containing eyes with the top and bottom quartiles of square-root GA growth rates.

### 2.7. Software

Analysis was conducted using python version 3.10.2, scipy 1.8.0, seaborn 0.11.2, scikit-learn 1.0.2, matplotlib 3.5.1, mrmr-selection 0.2.8, pandas 1.4.1, Pillow 9.0.1, and statsmodels 0.14.4.

## 3. Results

### 3.1. Radiomic Features Classifying ODS Phenotypes

**Figure 3:**
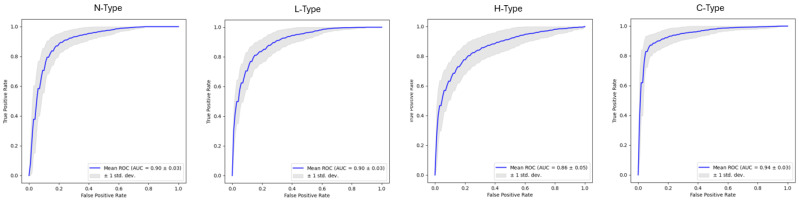
Classification of ODS phenotypes by quadratic discriminant analysis using drusen radiomic features.

**Figure 4:**
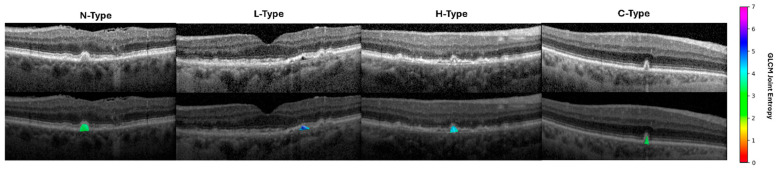
Visualization of a representative radiomic feature across drusen phenotypes. Heatmaps and color scale represent GLCM joint entropy value in the 10-pixel radius around each pixel within the druse. GLCM: gray level co-occurrence matrix.

Radiomic features classified ODS phenotypes with AUC = 0.90 +/− 0.03 for N-Type, 0.90 +/− 0.03 for L-Type, 0.86 +/− 0.05 for H-Type, and 0.94 +/− 0.03 for C-Type (Figure 3). The most important radiomic features included the drusen pixel intensity, texture, and shape (Table 1, Table 2, Table 3 and Table 4, Figure 4). Six important features were found for L-type ODSs, fifteen were found for H-type, four were found for C-type, and five were found for N-type (Table 1, Table 2, Table 3 and Table 4). L-type ODSs were characterized by texture and pixel intensity features: high NGTDM contrast, low 90th percentile pixel intensity, high GLSZM gray level variance, low kurtosis, and low pixel intensity uniformity (Table 1). H-type ODSs were characterized by texture and pixel intensity features, as well as shape elongation: high GLCM sum entropy, low radiomic elongation, high pixel intensity energy, high GLCM sum squares, high maximum pixel intensity, high GLCM autocorrelation, high GLCM joint entropy, low GLSZM size zone non-uniformity normalized, high pixel intensity total energy, high GLCM cluster tendency, high pixel intensity entropy, high GLDM dependence entropy, high GLDM high gray level emphasis, high GLCM correlation, and high pixel intensity range (Table 2). C-type ODSs were characterized by texture and shape features: low GLCM correlation, low minor axis length, low sphericity, and low GLCM sum entropy (Table 3). N-type ODSs were characterized by texture, shape, and pixel intensity features: high GLSZM large area emphasis, high sphericity, low pixel intensity entropy, low NGTDM contrast, and high GLSZM zone variance (Table 4).

### 3.2. Variation in Discriminating Radiomic Features Across ODS Type

**Figure 5:**
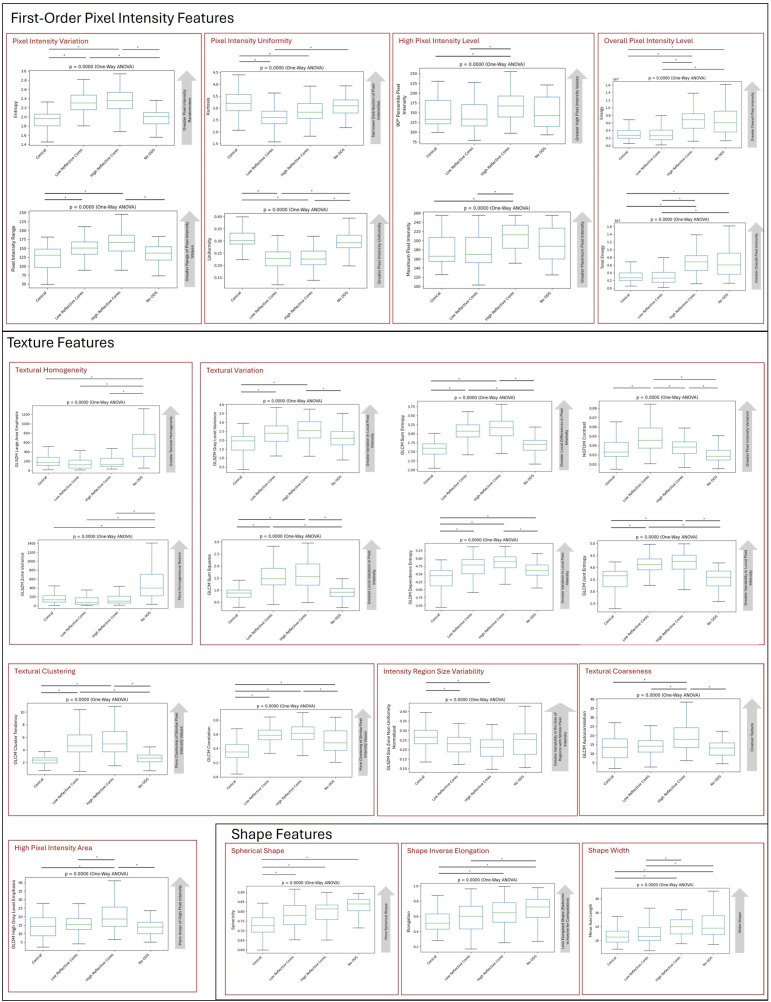
Variation in intensity, texture, and shape metrics by drusen substructure phenotype. Bars with asterisks above figure indicate statistical significance after multiple testing correction. Text in gray arrow is a plain-language interpretation of the radiomic feature. *: *p* < 0.05.

All discriminating features varied across ODS type (*p* < 0.00035) (Figure 5). The variation in important radiomic features is shown in Figure 5. First-order pixel intensity features showed a significant variation between individual OSD types (Figure 5). L-type and H-type ODSs had a higher pixel intensity variation (as shown by the higher entropy and range) and lower pixel intensity uniformity (as shown by kurtosis and uniformity) compared to C-type and N-type. H-type ODSs had higher high levels of pixel intensity, as evidenced by a greater 90th percentile and maximum intensity compared to C-type and L-type. H-type and N-type had higher overall levels of pixel intensity, as shown by a higher energy and total energy compared to C-type and L-type.

Texture features also showed a significant variation between individual ODS types (Figure 5). N-type ODSs had a greater textural homogeneity compared to C-type, L-type, and H-type, as shown by the increased GLSZM zone variance and GLSZM large area emphasis. L-type and H-type ODSs had a greater textural variation compared to C-type and N-type, as shown by the increased GLSZM gray level variance, GLCM sum squares, GLCM sum entropy, GLDM dependence entropy, NGTDM contrast, and GLCM joint entropy. L-type and H-type had an increased textural clustering compared to C-type and N-type, as shown by the increased GLCM cluster tendency and GLCM correlation. C-type ODSs showed an increased variability in the size of regions with similar pixel intensity, as shown by the increased GLSZM size zone non-uniformity compared to L-type and H-type. H-type had an increased textural coarseness compared to all other ODS types, as shown by the increased GLCM autocorrelation. H-type also had an increased high pixel intensity area compared to all other ODS types, as shown by the increased GLDM high gray level emphasis.

Shape features also varied significantly between individual ODS types (Figure 5). N-type had a more spherical shape than other ODS types, as shown by the increased sphericity. N-type and H-type ODSs were less elongated than L-type and C-type ODSs, as shown by the increased radiomic elongation (the inverse of true elongation). N-type and H-type ODSs also had a wider shape compared to C-type and L-type, as shown by the increased minor axis length.

### 3.3. Prediction of Geographic Atrophy Conversion and Growth with ODS Radiomics

**Figure 6:**
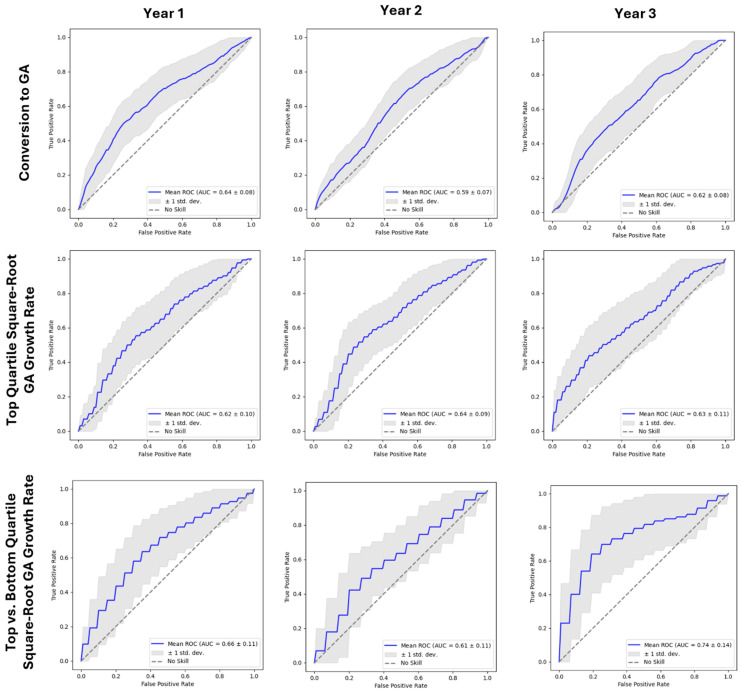
AUC curves of quadratic discriminant analysis classifiers predicting GA conversion and fast progression at years 1, 2, and 3. Dashed line indicates AUC of a no-skill classifier.

**Figure 7:**
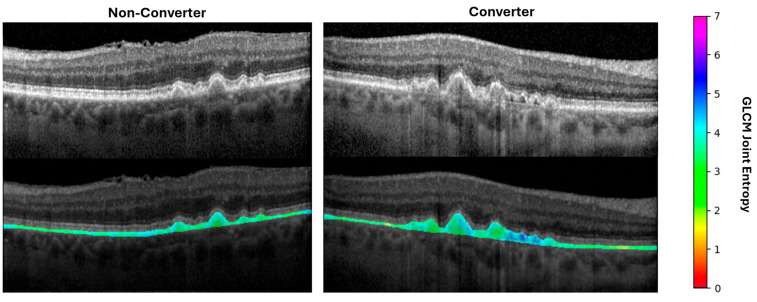
Variation in a representative radiomic feature between the RPE-BM compartments of eyes with iAMD that convert or do not convert to geographic atrophy within two years. Heatmaps and color scale represent GLCM joint entropy value in the 10-pixel radius around each pixel within the druse. GLCM: gray level co-occurrence matrix.

Radiomic features important for ODS classification extracted from the RPE-BM compartment predicted conversion to GA with AUC = 0.64 +/− 0.08, 0.59 +/− 0.07, and 0.62 +/− 0.08 at years 1, 2, and 3, respectively (Figure 6 and Figure 7). The features predicted top-quartile square-root GA growth rate with AUC = 0.62 +/− 0.10, 0.64 +/− 0.09, and 0.63 +/− 0.11 at years 1, 2, and 3, respectively (Figure 6 and Figure 7). Finally, the features predicted top-quartile square-root GA growth rate in a dataset of only eyes with top- and bottom-quartile square-root GA growth rate with AUC = 0.66 +/− 0.11, 0.61 +/− 11, and 0.74 +/− 0.14 at years 1, 2, and 3, respectively (Figure 6). All AUCs were significantly greater than the no-skill AUC of 0.5 (*p* < 0.0001). The mean AUC was significantly greater for predicting top-quartile versus bottom-quartile square-root GA growth rate compared to predicting top-quartile versus all other square-root GA growth rates at years 1 and 3 (*p* < 0.0001), but not year 2 (*p* = 0.12).

## 4. Discussion

These results demonstrate the potential utility of clinical phenotype-grounded interpretable radiomics-derived imaging signatures coupled with machine learning classification methods for image analysis and disease prognostication in dry AMD. This also supports the evidence-based theory that not all drusen are created equal, as well as the resulting potential for variable pathologic consequences on the retina, photoreceptors, and EZ. This work also demonstrates methods for the efficient and reliable automated quantification of drusen phenotypes with radiomics. As these radiomics were extracted from unaltered images across two device types, they reflect the shape and texture differences that are observed by clinicians regardless of imaging modality. A quadratic discriminant analysis of drusen radiomics enables the automated classification of ODS phenotypes with a high accuracy (AUC = 0.86–0.94), reflecting the suitability of radiomics for this classification task. The prediction of GA conversion and fast progression with these radiomics yielded AUC curves significantly greater than random chance, but not close to perfect prediction (AUC = 0.59–0.74), reflecting the partial role that ODS phenotypes play in the multifactorial nature of AMD progression.

Important radiomic features can be related to the characteristics of each ODS phenotype. L-type radiomics show a high textural variation, lower high-intensity regions, and lower overall pixel intensity, representing the texture of these drusen disrupted by low-reflective cores. Important radiomics for H-type ODSs show a high textural variation, coarse texture, high pixel intensity, and clustering texture, representing the high-reflective foci in these drusen. C-type ODSs show an elongated nonspherical shape with a low-level textural disorder, consistent with their conical shape and occasional internal debris. Finally, N-type radiomics show a relatively homogeneous radiomic texture, which is consistent with their appearance and definition.

Besides representing ODS definitions, radiomic features can reveal novel underlying differences between ODS phenotypes. For example, the number of radiomic features required for optimal ODS classification varied from 4 for C-type to 15 for H-type, reflecting possible differences in the underlying complexities of these phenotypes. C-type ODSs are consistently recognizable based on their unique conical shape and occasional internal debris, as shown by the shape and texture features that classify them. In contrast to the relatively consistent appearance of C-type ODSs, the large number of features describing H-type drusen suggests more complex or varied presentations of H-type ODSs—possibly including various sub-phenotypes of hyperreflective foci. This is consistent with the relatively decreased classification performance of radiomic features in discriminating H-type ODSs (AUC = 0.86) compared to other ODS types (AUC = 0.90–0.94). The existence of such sub-phenotypes and possible implications for underlying pathophysiology should be investigated further, especially as the histological composition of drusen is known to be heterogeneous [21].

Beyond revealing novel underlying characteristics, this radiomic analysis suggests the interplay between shape and texture features of ODSs in ways that have not to our knowledge been investigated. While N-type ODSs are defined by internal texture, this analysis noted the relatively spherical shape of N-type compared to other ODSs. Similarly, H-type ODSs are defined by their internal texture but were noted to have a more wide and spherical shape. As the pathogenesis of AMD likely involves many biomolecular processes with varying impacts on anatomy, the relationships between the different imaging characteristics of ODSs merits further investigation.

This study has notable advantages, including semi-automated OCT segmentation with humans in the loop, quantitative radiomic retinal compartment analysis, and a large clinical database of images. These results build on previous work in automated ophthalmic image processing and present opportunities for future clinical utility. Given the recent approval of machine learning-assisted EZ loss segmentation as a clinical trial endpoint [4], as well as the long-standing use of drusen as an AMD biomarker, it is reasonable to propose machine learning-assisted analysis of drusen as a clinical measure of the disease state or a clinical trial endpoint. Such measures would require further investigation and validation to control for any variation in image characteristics due to the device or image quality, but could be valuable, especially if future therapeutics target drusen formation.

This study has several important limitations. While this study examined 104 radiomic features, other radiomic features may exist that were not evaluated, which may be relevant to ODS phenotypes. Additionally, techniques such as image filtering, intensity normalization, etc., prior to radiomics extraction may improve the classifier performance, albeit possibly at the expense of clinical interpretability and relatedness to what clinicians directly observe across imaging modalities. Isolating drusen from the RPE-BM compartment with image processing methods could be further used to improve the signal-to-noise ratio and possibly improve GA prediction. These techniques should be investigated further. Additionally, as a solely imaging-based study, these ODS radiomics could not be correlated to the underlying molecular biology of AMD. This too is important for future work.

In summary, this study demonstrated clinical phenotype-grounded radiomic analysis as a useful technique for automated drusen classification and disease prognostication in dry AMD. The results revealed interpretable findings that both reflected the known clinical phenotypes and revealed novel information about the complexity of ODS phenotypes and the interplay of ODS texture and shape. While further investigation is needed to validate these measures in clinical trials and clinical practice settings, these results are a proof of feasibility for clinical phenotype-grounded radiomics in dry AMD.

## Figures and Tables

**Table 1:** Most important discriminating radiomics for L-type drusen.

ODS Type	Best Discriminating Features	Median Value Relative to Other ODS Types	Feature Interpretation	AUC
L-type: Low-reflective cores	NGTDM Contrast	High	High local pixel intensity variation	0.90 +/− 0.03
	First-Order 90th Percentile	Low	Brightest pixels within the druse have low pixel intensity	
	GLSZM Gray Level Variance	High	High variation in local pixel intensity	
	First-Order Kurtosis	Low	Less narrow distribution of pixel intensity values	
	First-Order Uniformity	Low	Low pixel intensity uniformity	
	First-Order Energy	Low	Low overall pixel intensity	

GLSZM: gray level size zone matrix, NGTDM: neighboring gray tone difference matrix.

**Table 2:** Most important discriminating radiomics for H-type drusen.

ODS Type	Best Discriminating Features	Median Value Relative to Other ODS Types	Feature Interpretation	AUC
H-type: High-reflective cores	GLCM Sum Entropy	High	Large local differences in pixel intensity	0.87 +/− 0.05
	Elongation (Radiomic elongation is inverse of true elongation for computational reasons)	Low	Low elongation	
	First-Order Energy	High	High overall pixel intensity	
	GLCM Sum Squares	High	High local variation in pixel intensity	
	First-Order Maximum	High	High maximum pixel intensity	
	GLCM Autocorrelation	High	Coarser texture	
	GLCM Joint Entropy	High	High variability in local pixel intensity	
	GLSZM Size Zone Non-Uniformity Normalized	Low	Low variability in the size of regions with similar intensity	
	First Order Total Energy	High	High overall pixel intensity	
	GLCM Cluster Tendency	High	Pixels with similar intensity tend to cluster together	
	First Order Entropy	High	High variation in pixel intensity	
	GLDM Dependence Entropy	High	High variation in local pixel intensity	
	GLDM High Gray Level Emphasis	High	Many areas of high pixel intensity	
	GLCM Correlation	High	Pixels with similar intensity tend to cluster together	
	First-Order Range	High	High variability of pixel intensity	

GLDM: gray level dependence matrix, GLSZM: gray level size zone matrix, GLCM: gray level co-occurrence matrix.

**Table 3:** Most important discriminating radiomics for C-type drusen.

ODS Type	Best Discriminating Features	Median Value Relative to Other ODS Types	Feature Interpretation	AUC
C-type: Conical	GLCM Correlation	Low	High randomness in local pixel intensity values	0.95 +/− 0.03
	Minor Axis Length	Low	Narrow shape	
	Sphericity	Low	Less spherical shape	
	GLCM Sum Entropy	Low	Small local differences in pixel intensity	

GLCM: gray level co-occurrence matrix.

**Table 4:** Most important discriminating radiomics for N-type drusen.

ODS Type	Best Discriminating Features	Median Value Relative to Other ODS Types	Feature Interpretation	AUC
N-type: no substructure—homogeneous reflectivity	GLSZM Large Area Emphasis	High	More homogenous texture	0.90 +/− 0.03
	Sphericity	High	More spherical shape	
	First-Order Entropy	Low	Low randomness of intensity values	
	NGTDM Contrast	Low	Low local pixel intensity variation	
	GLSZM Zone Variance	High	Some very large areas of similar pixel intensity, as well as some very small regions of similar pixel intensity	

GLSZM: gray level size zone matrix, NGTDM: neighboring gray tone difference matrix.

## Data Availability

The original contributions presented in this study are included in the article. Further inquiries can be directed to the corresponding author.

## Abbreviations

The following abbreviations are used in this manuscript:

ODS	Optical coherence tomography-reflective drusen substructure
OCT	Optical coherence tomography
GA	Geographic atrophy
AMD	Age-related macular degeneration
AUC	Area under the receiver operating characteristic curve
SD-OCT	Spectral domain optical coherence tomography
FDA	United States Food and Drug Administration
EZ	Ellipsoid zone
RPE	Retinal pigment epithelium
BM	Bruch’s membrane
IRB	Institutional Review Board
ANOVA	Analysis of variance
GLDM	Gray level dependence matrix
GLRLM	Gray level run length matrix
GLSZM	Gray level size zone matrix
GLCM	Gray level co-occurrence matrix
NGTDM	Neighboring gray tone difference matrix

## References

[1] Girgis S., Lee L.R. (2023). Treatment of Dry Age-Related Macular Degeneration: A Review. Clin. Exp. Ophthalmol..

[2] Papadopoulos Z. (2020). Recent Developments in the Treatment of Wet Age-Related Macular Degeneration. Curr. Med. Sci..

[3] Lad E.M., Finger R.P., Guymer R. (2023). Biomarkers for the Progression of Intermediate Age-Related Macular Degeneration. Ophthalmol. Ther..

[4] Ehlers J.P., Hu A., Boyer D., Cousins S.W., Waheed N.K., Rosenfeld P.J., Brown D., Kaiser P.K., Abbruscato A., Gao G. (2025). ReCLAIM-2: A Randomized Phase II Clinical Trial Evaluating Elamipretide in Age-Related Macular Degeneration, Geographic Atrophy Growth, Visual Function, and Ellipsoid Zone Preservation. Ophthalmol. Sci..

[5] Trinh M., Cheung R., Duong A., Nivison-Smith L., Ly A. (2024). OCT Prognostic Biomarkers for Progression to Late Age-Related Macular Degeneration: A Systematic Review and Meta-Analysis. Ophthalmol. Retin..

[6] Yaqoob Z., Wu J., Yang C. (2005). Spectral Domain Optical Coherence Tomography: A Better OCT Imaging Strategy. BioTechniques.

[7] Itoh Y., Vasanji A., Ehlers J.P. (2016). Volumetric Ellipsoid Zone Mapping for Enhanced Visualisation of Outer Retinal Integrity with Optical Coherence Tomography. Br. J. Ophthalmol..

[8] Spaide R.F., Curcio C.A., Zweifel S.A. (2010). Drusen, an Old but New Frontier. Retina.

[9] Wang L., Clark M.E., Crossman D.K., Kojima K., Messinger J.D., Mobley J.A., Curcio C.A. (2010). Abundant Lipid and Protein Components of Drusen. PLoS ONE.

[10] Veerappan M., El-Hage-Sleiman A.-K.M., Tai V., Chiu S.J., Winter K.P., Stinnett S.S., Hwang T.S., Hubbard G.B., Michelson M., Gunther R. (2016). Optical Coherence Tomography Reflective Drusen Substructures Predict Progression to Geographic Atrophy in Age-Related Macular Degeneration. Ophthalmology.

[11] Scapicchio C., Gabelloni M., Barucci A., Cioni D., Saba L., Neri E. (2021). A Deep Look into Radiomics. Radiol. Med..

[12] Bera K., Braman N., Gupta A., Velcheti V., Madabhushi A. (2022). Predicting Cancer Outcomes with Radiomics and Artificial Intelligence in Radiology. Nat. Rev. Clin. Oncol..

[13] Corredor G., Bharadwaj S., Pathak T., Viswanathan V.S., Toro P., Madabhushi A. (2023). A Review of AI-Based Radiomics and Computational Pathology Approaches in Triple-Negative Breast Cancer: Current Applications and Perspectives. Clin. Breast Cancer.

[14] Kar S.S., Cetin H., Abraham J., Srivastava S.K., Whitney J., Madabhushi A., Ehlers J.P. (2023). Novel Fractal-Based Sub-RPE Compartment OCT Radiomics Biomarkers Are Associated with Subfoveal Geographic Atrophy in Dry AMD. IEEE Trans. Biomed. Eng..

[15] Kar S.S., Cetin H., Lunasco L., Le T.K., Zahid R., Meng X., Srivastava S.K., Madabhushi A., Ehlers J.P. (2022). OCT-Derived Radiomic Features Predict Anti–VEGF Response and Durability in Neovascular Age-Related Macular Degeneration. Ophthalmol. Sci..

[16] Sil Kar S., Sevgi D.D., Dong V., Srivastava S.K., Madabhushi A., Ehlers J.P. (2021). Multi-Compartment Spatially-Derived Radiomics From Optical Coherence Tomography Predict Anti-VEGF Treatment Durability in Macular Edema Secondary to Retinal Vascular Disease: Preliminary Findings. IEEE J. Transl. Eng. Health Med..

[17] Kar S.S., Cetin H., Abraham J., Srivastava S.K., Madabhushi A., Ehlers J.P. (2024). Combination of Optical Coherence Tomography-Derived Shape and Texture Features Are Associated with Development of Sub-Foveal Geographic Atrophy in Dry AMD. Sci. Rep..

[18] Reyes M., Meier R., Pereira S., Silva C.A., Dahlweid F.-M., von Tengg-Kobligk H., Summers R.M., Wiest R. (2020). On the Interpretability of Artificial Intelligence in Radiology: Challenges and Opportunities. Radiol. Artif. Intell..

[19] Van Griethuysen J.J.M., Fedorov A., Parmar C., Hosny A., Aucoin N., Narayan V., Beets-Tan R.G.H., Fillion-Robin J.-C., Pieper S., Aerts H.J.W.L. (2017). Computational Radiomics System to Decode the Radiographic Phenotype. Cancer Res..

[20] Ding C., Peng H. (2003). Minimum Redundancy Feature Selection from Microarray Gene Expression Data. Proceedings of the Computational Systems Bioinformatics. CSB2003. Proceedings of the 2003 IEEE Bioinformatics Conference. CSB2003.

[21] Litts K.M., Zhang Y., Freund K.B., Curcio C.A. (2018). Optical Coherence Tomography and Histology of Age-Related Macular Degeneration Support Mitochondria as Reflectivity Sources. Retina.

